# Intrahepatic Mass-Forming Cholangiocarcinoma: Is There Additional Prognostic Value in Using Gd-EOB Enhanced MRI?

**DOI:** 10.3390/cancers16071314

**Published:** 2024-03-28

**Authors:** Sebastian Halskov, Felix Krenzien, Laura Segger, Dominik Geisel, Bernd Hamm, Uwe Pelzer, Jana Ihlow, Wenzel Schöning, Timo Alexander Auer, Uli Fehrenbach

**Affiliations:** 1 Department of Radiology, Charité—Universitätsmedizin Berlin, Augustenburger Platz 1, 13353 Berlin, Germany; 2 Berlin Institute of Health, Anna-Louisa-Karsch-Straße 2, 10178 Berlin, Germany; 3 Department of Surgery, Charité—Universitätsmedizin Berlin, Augustenburger Platz 1, 13353 Berlin, Germany; 4 Department of Hematology, Oncology and Cancer Immunology, Charité—Universitätsmedizin Berlin, Augustenburger Platz 1, 13353 Berlin, Germany; 5 Institute of Pathology, Charité—Universitätsmedizin Berlin, Charitéplatz 1, 10117 Berlin, Germany

**Keywords:** gadoxetic acid, cholangiocellular carcinoma, intrahepatic mass-forming cholangiocarcinoma, liver, magnetic resonance imaging

## Abstract

**Simple Summary:**

Cholangiocarcinomas are aggressive tumors that arise from the biliary system. They are highly diverse, both morphologically, in terms of histology and imaging, and biologically in terms of prognosis and response to therapy. Robust predictors of prognosis are critical to ensure that the most effective therapy is allocated to each patient. The presence of a dense, fibrotic stroma within the tumor has already been associated with a poor prognosis in mass-forming intrahepatic cholangiocarcinoma (IMCC). This histologic feature has also been associated with increased retention of gadoxetic acid (Gd-EOB), a liver-specific contrast agent in magnetic resonance imaging (MRI). We investigated whether the IMCC’s signal intensity in the late phase of Gd-EOB-enhanced MRI can non-invasively identify patients who have a relatively worse prognosis.

**Abstract:**

Objective: To investigate the prognostic value of enhancement patterns of intrahepatic mass-forming cholangiocarcinomas (IMCCs) during the hepatobiliary phase (HBP) in gadoxetic acid (Gd-EOB)-enhanced MRI. Methods: We retrospectively identified 66 consecutive patients with histopathologically proven IMCCs (reference standard: resection) and preoperative Gd-EOB-enhanced MRI. Gd-EOB retention area was subjectively rated based on areas of intermediate signal intensity. Lesions were classified as either hypointense (0–25% retention area) or significantly-retaining (>25% retention area). Clinical, radiological, and prognostic features were compared between these groups. The primary endpoints were recurrence-free survival (RFS) and overall survival (OS) after primary surgical resection. Results: 73% (48/66) of lesions were rated as hypointense and 29% (19/66) as significantly-retaining. While the hypointense subgroup more frequently featured local and distant intrahepatic metastases (*p* = 0.039 and *p* = 0.022) and an infiltrative growth pattern (*p* = 0.005), RFS, OS, and clinical features did not differ significantly with estimated Gd-EOB retention area or quantitatively measured HBP enhancement ratios. Lymph node metastasis was an independent predictor of poor RFS (*p* = 0.001). Conclusions: Gd-EOB-enhanced MRI revealed two subtypes of IMCC in the HBP: hypointense and signal-retaining. The hypointense subtype is associated with more frequent intrahepatic metastases and an infiltrative growth pattern, indicating potential tumor aggressiveness. However, this did not result in a significant difference in survival after the primary resection of IMCC.

## 1. Introduction

Worldwide, cholangiocellular carcinoma (CCA) accounts for 3% of all gastrointestinal cancers and arises from the ductal epithelium of the biliary tree [1]. CCAs can further be classified based on localization into intrahepatic, perihilar, distal, and gallbladder carcinomas [2,3]. Intrahepatic CCAs are rare but account for 10–15% of all primary liver cancers and represent the most common primary non-hepatocellular carcinoma (HCC) malignancy in the non-cirrhotic liver [1,4]. The most common type is intrahepatic mass-forming cholangiocellular carcinoma (IMCC), which is associated with a dismal prognosis. Surgery at an early stage remains the only curative treatment option [3,4,5,6].

Similarly to HCC, IMCC exhibits very high heterogeneity in histopathology, imaging, and prognosis, which could result from diverse cellular origins [7,8,9,10]. A histopathological subclassification has been proposed and adopted by the WHO in the 5th edition of its Classification of Digestive System Tumors, which divides IMCC into a small-duct, cholangiolar type associated with mutations of isocitrate dehydrogenase and a large-duct type, which resembles extrahepatic CC and is associated with a worse prognosis, lymph node metastasis, and KRAS mutations [10,11,12]. On cross-sectional imaging, the large-duct type has been associated with the absence of arterial phase hyperenhancement (APHE), an infiltrative appearance, vascular invasion, and diffuse biliary dilatation [13,14]. Further groups have established the arterial phase enhancement pattern to be an independent predictor of survival following primary surgical resection in MRI [15,16] and CT [17,18].

Given the considerable heterogeneity of CCAs, a full characterization of each tumor phenotype is essential for making accurate predictions of each patient’s prognosis for optimized therapeutic strategies. In pretherapeutic diagnostic workup, magnetic resonance imaging (MRI) is a powerful tool that is non-invasive and repeatable and offers a multiparametric analysis of lesions with excellent soft-tissue contrast. In the context of IMCC, MRI can leverage that a dense, fibrous stroma is a feature of large-duct IMCC [10] and has been associated with specific imaging features. Yamada et al. demonstrated that a higher degree of diffusion restriction was associated with a rich fibrous stroma and a worse prognosis [19]. Furthermore, hepatobiliary contrast agents such as gadoxetic acid (Gd-EOB) are retained to a greater extent by densely fibrotic IMCCs in the hepatobiliary phase (HBP), resulting in a higher signal intensity that could be used to identify lesions that carry a poor prognosis [20].

MRI also has inherent advantages as a non-invasive modality. While biopsy can provide a wealth of prognostic information that is essential for initiating systemic therapy, it exposes the patient to additional risks. In percutaneous liver biopsy, these include bleeding, pain, hospitalization, and, potentially, needle tract seeding [21,22]. Furthermore, not all tumor locations may be readily accessible. There are thus divergent recommendations for biopsy in national guidelines for potentially resectable IMCC [23]. We believe imaging-based prognosticators may be more readily integrated into existing diagnostic workups for potentially resectable IMCC.

HBP morphology could thus be of additional prognostic value in IMCC but is not well studied in this context. Survival analysis by Koh et al. found a shorter survival time and time to recurrence in multivariate analysis of signal-retaining lesions [20]. Conversely, Kang et al. found that signal-retaining IMCCs were overall better differentiated and had lower rates of lymph node metastasis [8], suggesting a better prognosis [24].

The purpose of this study was therefore to investigate the prognostic value of imaging morphology in Gd-EOB-enhanced MRI, particularly the influence of HBP signal intensity on postresection survival and rate of complications.

## 2. Materials and Methods

### 2.1. Patients

We retrospectively identified patients from a prospectively maintained surgical database of consecutive patients. The inclusion criteria were as follows:

- Histopathologically proven IMCC following surgical resection between 1 January 2011 and 31 December 2019 at the Department of Surgery, Charité—Universitätsmedizin Berlin.
- Preoperative abdominal Gd-EOB-enhanced MRI.
- Mass-forming growth pattern.

The exclusion criteria were as follows:

- Insufficient quality of preoperative imaging (e.g., severe motion artifacts in HBP).
- Histopathology other than IMCC (e.g., mixed HCC-CCA).

### 2.2. Clinical Features

Clinical data relating to prognosis were recorded. These included data on serum tumor markers, TNM classification, histological grade according to Edmondson and Steiner, postoperative complications, the duration of the postoperative stay in hospital, the length of recurrence-free survival (RFS), and overall survival (OS) after primary surgical resection. RFS was defined as ending with recurrence or death, while OS ended with death. Follow-up at our center consisted of regular physical, laboratory, and imaging examinations starting 6 to 8 weeks after surgery and then at 3- to 6-month intervals.

### 2.3. Imaging

Our standard imaging protocols for MRI were carried out with phased-array body coils at 1.5 or 3 T. Precontrast sequences were obtained in T1- and T2-weighting and were both repeated with fat saturation, while T1w sequences included in-/opposed-phase technique. Diffusion-weighted imaging (DWI) was obtained at B-values of 50, 400, and 800. All sequences except DWI were obtained in breath-hold. Gd-EOB was administered intravenously at 0.025 mmol/kg body weight, with manual or automatic injection at a flow rate of 1–2 mL/s followed by a 40 mL saline flush. Subsequently, multiphase T1w sequences with FS were obtained with the following fixed delays: arterial phase—15 s, portal venous phase—50 s, transitional phase—90 s, and hepatobiliary phase—20 min. Further details of our standard protocol at 1.5 T may be found in Supplementary Table S1.

### 2.4. Readers

All images were read in consensus by two board-certified radiologists blinded to the clinical data and with expertise in liver imaging: T.A.A.: 7 years of experience; U.F.: 10 years of experience.

### 2.5. Analysis of HBP Gd-EOB Retention

**Figure 1:**
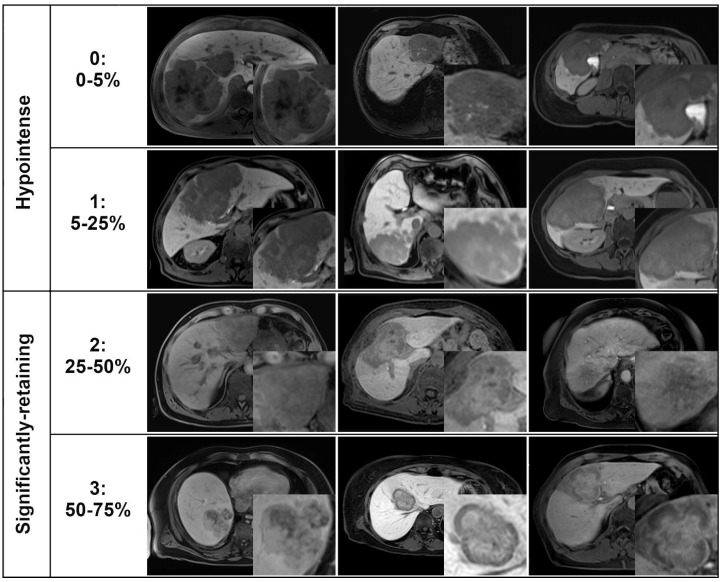
Rating scale for qualitative scoring of Gd-EOB retention area based on the visually estimated % of the lesion’s area showing near isointensity to hyperintensity relative to liver parenchyma in the HBP. Each row corresponds to one score, with the quoted percentage indicating the estimated % of the lesion area, and 3 example lesions from the study cohort that have been assigned this score. The highest score of 4 (75–100%) is not shown because no lesions were assigned this score.

Gd-EOB retention area was rated by our readers based on the estimated portion of the lesion’s area at its maximal diameter showing near isointensity to hyperintensity in comparison to adjacent liver parenchyma. A 5-point scale was used: 0, 0–5%; 1, 5–25%; 2, 25–50%; 3, 50–75%; 4, 75–100%. This closely matches the method described in previous studies of focal liver lesions [25,26]. Lesions with Gd-EOB uptake scores of 0–1 were classified as hypointense, and lesions with scores of 2–4 were classified as significantly-retaining (Figure 1).

For the quantitative assessment of HBP enhancement, polygonal 2D ROIs including the entire tumor at its maximum cross-sectional diameter were placed manually by L.S.S. (3 years of experience). All ROIs were placed in the HBP and cloned to the precontrast phase. An additional circular ROI with a fixed diameter of 10 mm was placed in healthy liver parenchyma without including vessels and bile ducts. The lesion-to-liver signal enhancement ratio was calculated as follows:

100 × (lesion signal enhancement/liver signal enhancement)

### 2.6. Qualitative Imaging Features

The qualitative parameters recorded in this study are defined in Table 1, using the LI-RADS lexicon as a foundation for defining imaging features [27].

### 2.7. Statistical Analysis

Statistical analysis was performed with XLSTAT (version 2011.0.01; Addinsoft SARL, New York, NY, USA) and SPSS software (version 29.0.0.0; IBM, Armonk, NY, USA). Descriptive statistical analysis was performed for all variables. The distribution of categorical variables was analyzed using the Chi-squared and Fisher’s exact test. For continuous variables, normal distribution was not assumed based on histograms and quantile plots. Therefore, the Mann–Whitney U test was used to compare central tendencies between groups. Survival rates were visualized by means of Kaplan–Meier curves, using a log-rank function to test for statistically significant differences. Multivariate Cox regression analysis was performed for multivariate analysis of potential predictors of survival. Inter-reader variability was tested using Cohen’s kappa test, and additionally Kendall’s tau for ordinal variables. The agreement was rated as follows: к = 0.0–0.20: none, 0.21–0.39: minimal, 0.40–0.59: weak, 0.60–0.79: moderate, 0.80–0.90 strong, and above 0.90: almost perfect [28]. Patients lost to follow-up were censored. Observations that were missing or recorded with uncertainty were removed from the analysis. A statistically significant difference was assumed for variables with a two-sided *p*-value less than 0.05.

## 3. Results

### 3.1. Selection Procedure

Out of 250 patients extracted from the surgical database for inclusion, 66 met the inclusion criteria for our analysis. The median interval between preoperative imaging and primary resection was 17 days (7–34).

### 3.2. Radiological Features

The results of qualitative and quantitative imaging analysis are shown in Table 2. A total of 47 lesions were assigned to the hypointense group, most of which had a Gd-EOB retention area score of 1 (81%, 38/47). A total of 19 lesions were assigned to the significantly-retaining group, where a score of 3 was the most common (58%, 11/19). Inter-reader agreement for classifying retention area was moderate, with a Cohen’s kappa of 0.61 (95% confidence interval: 0.39–0.82) for subdivision into the hypointense and significantly-retaining groups. The quantitatively measured HBP enhancement ratio was significantly higher in the significantly-retaining group (median 0.70, 0.54–0.89 compared to 0.47, 0.39–0.63) (*p* = 0.002 *).

**Figure 2:**
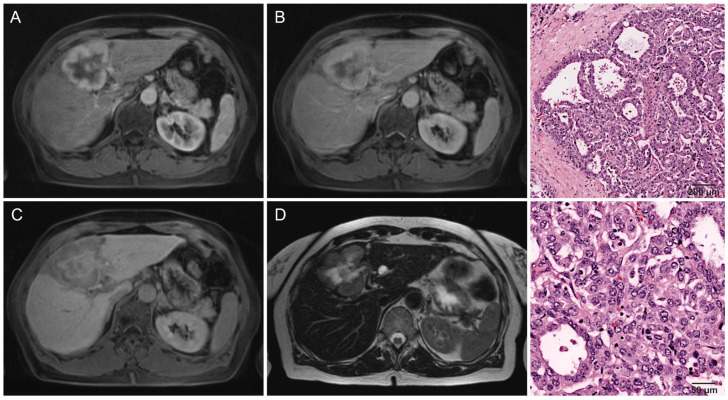
A 70-year-old woman with liver fibrosis, Desmet grade I. Hypervascular IMCC in segment IVa/IVb without vascular invasion or lymph node metastasis, with no recurrence registered after 550 days of follow-up. The lesion shows (**A**) rim APHE, (**B**) portal venous phase washout, and (**C**) a central area of isointensity relative to liver parenchyma surrounded by a hypointense rim, giving it a Gd-EOB retention area score of 3 (50–75%). (**D**) T2w-imaging shows central necrosis (high signal) with surrounding fibrous tissue (low signal). (**Right column**) Histological images of a lesion with similar imaging features, with increasing magnification. Intrahepatic cholangiocarcinoma in otherwise healthy liver parenchyma. The overall growth pattern is consistent with a large-duct type. The tumor shows a tubuloglandular architecture and consists of large glands lined by tall columnar cells.

**Figure 3:**
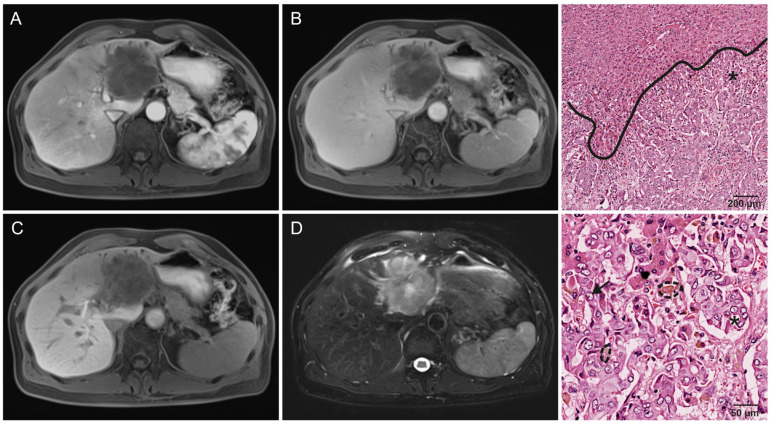
A 52-year-old man without liver cirrhosis who underwent primary surgical resection for IMCC with lymphatic node metastasis and microvascular invasion, which recurred after 420 days. (**A**) IMCC in the left liver lobe without arterial phase enhancement and (**B**) with minimal enhancement until the delayed phase. (**C**) The lesion is almost entirely hypointense in HBP, giving it a Gd-EOB retention area score of 0. (**D**) T2w-imaging with FS shows a small central area of necrosis (high signal) without clear demarcation of fibrous stroma. (**Right column**) Histological images of a lesion with similar imaging features, with increasing magnification. Intrahepatic cholangiocarcinoma in otherwise healthy liver parenchyma. An asterisk marks the tumor. The predominant growth pattern is consistent with a small-duct type. The tumor consists of ductular cord-like glands with hyalinized fibrous stroma. The tumor cells show an increased nuclear/cytoplasm ratio and prominent intranuclear nucleoli (arrow). Adjacent to the tumor, there is bile pigment as a correlate for cholestasis (encircled by dashed lines).

**Figure 4:**
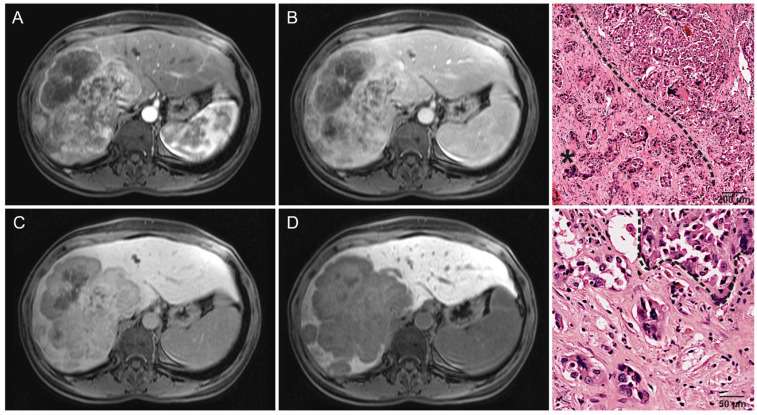
A 50-year-old woman with no signs of chronic liver disease. Surgically resected IMCC with microvascular invasion, positive resection margins, local intrahepatic metastases, and no lymphonodal metastasis. Recurrence was observed after 293 days. The right-hepatic lesion is notable for exhibiting two distinct areas of enhancement: (**A**) a ventral portion with pseudocapsule enhancement and a dorsal portion with diffuse inhomogeneous APHE. (**B**) Washout occurs in the portal venous phase, (**C**) and a hypoenhancing central area surrounded by a progressively enhancing rim becomes apparent within the ventral portion in the delayed phase. (**D**) The lesion is nearly uniformly hypointense in HBP, resulting in a Gd-EOB retention area score of 0. (**Right column**) Histological images of this lesion, with increasing magnification. Intrahepatic cholangiocarcinoma exhibiting both features of a small-duct type and a large-duct type. An asterisk marks the small-duct component.

There was no significant difference in early enhancement patterns, the most common being progressive enhancement in approximately half of all lesions (*p* = 0.890). A total of 33% (21/64) of lesions were hypervascular (Figure 2), while 14% (9/64) showed minimal enhancement (Figure 3). A single lesion clearly showed two distinct areas of enhancement, but histopathological analysis showed this tumor to be monophenotypic (Figure 4). Washout was uncommon in both groups (18%, 8/45 and 27%, 5/19) (*p* = 0.027), with a significant difference due to non-peripheral washout being more frequent in hypointense IMCC. All hypointense lesions were lobulated, while 11% (2/19) of significantly-retaining lesions had a round shape (*p* = 0.022). Significantly-retaining lesions were more likely to have sharp margins (53%, 10/19 compared to 17%, 8/47) (*p* = 0.006) and a solid growth pattern (68%, 13/19 compared to 31%, 15/48) (*p* = 0.005). Intrahepatic metastasis was considerably less frequent in the significantly-retaining group, both the local (26%, 5/19 compared to 53%, 25/47) (*p* = 0.039) and distant type (0%, 0/19 compared to 23%, 11/47) (*p* = 0.022). No other statistically significant associations were found (*p* ≥ 0.05).

### 3.3. Clinical Features

Clinical features are summarized in Table 3. Approximately half of the patients were male in both the hypointense (45%, 21/47) and significantly-retaining groups (53%, 10/19) (*p* = 0.616). The median age was 63 (55–70) and 69 years (58–77), respectively, with no significant difference (*p* = 0.068). The most common histological grade, according to Edmondson–Steiner, in both groups, was 2 (72%, 33/46 and 67%, 12/18), while grade 1 was only assigned to two hypointense lesions (4%, 2/46) (*p* = 0.865). The majority of IMCCs were resected in the T1 or T2 stages according to the TNM classification (85%, 39/46 and 68%, 13/19) (*p* = 0.305), with no significant differences in T, N, V, or R stages between groups.

Postoperative complications occurred in 72% (31/43) of cases in the hypointense group and 88% (15/17) of cases in the significantly-retaining group (*p* = 0.311), with the most common being bile leakage that required therapeutic intervention (19%, 8/43 and 29%, 5/17) (*p* = 0.486). The median duration of stay in an intensive care unit (2 [1–3] and 1 [1–5] days, *p* = 0.617) and a hospital ward (13 [8–24] and 18 [13–49] days, *p* = 1.000) did not differ significantly.

**Figure 5:**
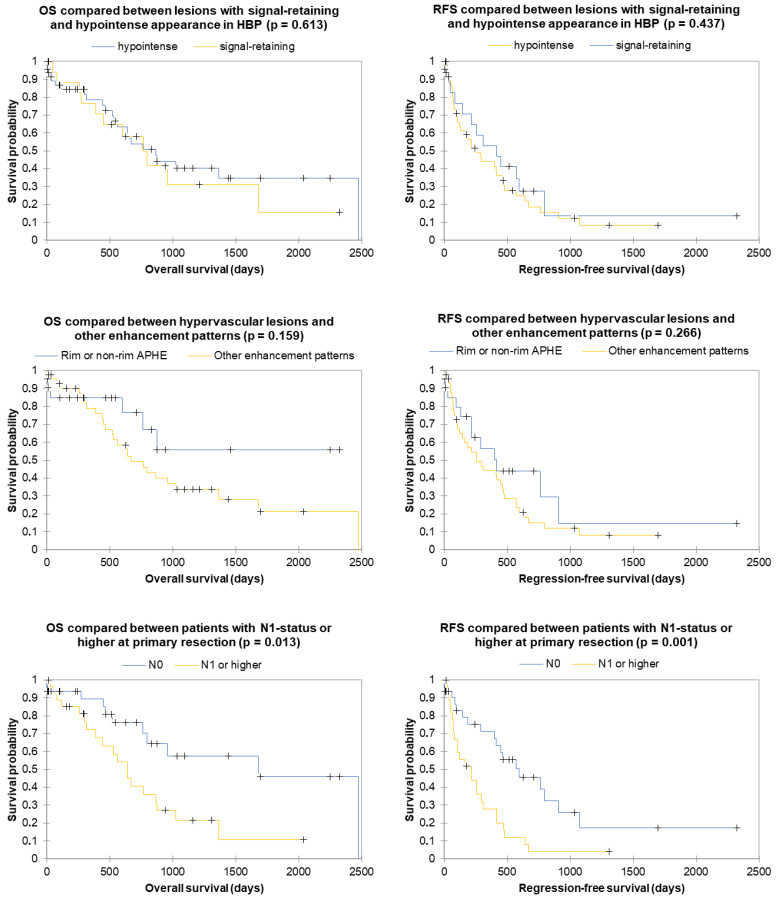
Kaplan–Meier curves to visualize the probability of overall survival (**left column**) and recurrence-free survival (**right column**) following primary resection as a function of time. Comparisons have been made between subgroups of patients with (**top row**) N0 or N1 and higher status, (**middle row**) lesions with hypervascular or other enhancement patterns, and (**bottom row**) lesions with hypointense or significantly-retaining appearance in HBP. Crosses mark censored events. The *p*-value represents the result of a log-rank comparison between the curves of both subgroups.

The median duration of follow-up was 518 (154–1058) days. Recurrences were observed in 57% (27/47) and 42% (8/19) of cases, while deaths were observed in 49% (23/47) and 58% (11/19) of cases. Kaplan–Meier curves visualizing OS and RFS are shown in Figure 5 for comparing the prognostic impact of N1 status or higher and hypervascular and significantly-retaining appearance. A significant difference was found for both OS and RFS regarding N1 status (*p* = 0.013 and *p* = 0.001) but not for hypervascular (*p* = 0.159 and *p* = 0.266) or significantly-retaining (*p* = 0.613 and *p* = 0.437) appearance. The results of multivariate Cox regression analysis for the prediction of OS and RFS by qualitative and quantitative markers of HBP enhancement, in combination with previously reported radiological and postresection histopathological predictors of survival [29,30], are displayed in Table 4. Notably, HBP morphology did not reach statistical significance, while N1 status or higher was a predictor of poor RFS (2.85 (1.50–5.40), *p* = 0.001) and OS (1.73 (0.79–3.79), *p* = 0.004), and intrahepatic metastasis was a predictor of poor RFS (2.53 (1.34–4.75), *p* = 0.004).

## 4. Discussion

We retrospectively analyzed the clinical, radiological, and histopathological data of 66 patients with IMCCs resected at our center. Lesions were qualitatively and quantitatively assessed for their enhancement in HBP and subsequently subclassified into hypointense and significantly-retaining groups for comparison. We found statistically significant associations with other radiological imaging features, including some that have been associated with postresection survival. However, this did not translate into differences in survival or complication rates in our cohort.

The majority of statistically significant associations with the Gd-EOB retention area variable were found with other radiological imaging features. This was most notably the case for local and distant intrahepatic metastasis, both of which were less common in significantly-retaining IMCCs. As intrahepatic metastasis was associated with a shorter RFS and OS in our cohort and a meta-analysis by Mavros et al. [24], this would suggest a better prognosis for patients with significantly-retaining IMCCs. However, statistical analysis of our data regarding perioperative complications, histopathological properties, and survival did not support a difference in prognosis on the basis of HBP morphology, even after taking into consideration the comparatively small size of the study sample for this uncommon tumor entity. An alternative explanation for the higher detection rate of intrahepatic metastasis in poorly enhancing lesions could be higher conspicuity relative to liver parenchyma, as previously demonstrated by Kang et al. [8].

We found an association of lymph node metastasis at primary surgical resection—a typical feature of large-duct IMCC [11]—with poorer RFS (*p* = 0.001) and OS (*p* = 0.013). We also showed that patients with hypervascular IMCC had improved RFS and OS, albeit without reaching the threshold for statistical significance in this cohort (*p* = 0.266 and *p* = 0.159, respectively). These are important findings for indicating the external validity of our results.

Previous studies have so far presented a mixed picture regarding the prognostic impact of higher HBP enhancement. Koh et al. demonstrated a shorter survival time and time to recurrence on multivariate analysis for such lesions [20]. Conversely, Kang et al. found that signal-retaining IMCCs had better differentiation and lower rates of lymph node metastasis [8], factors which have in turn been associated with a better prognosis [24]. This heterogeneity could partly be explained by the retrospective nature of both studies. Furthermore, Kang et al. quantified HBP enhancement using circular 2D ROIs, while Koh et al. visually estimated the portion of the lesion area that showed intermediate signal intensity, similar to our Gd-EOB retention area score. While we found a strong association between our visual estimates and the central tendencies of quantitatively measured HBP enhancement ratios (*p* = 0.002), this could still represent a key methodological difference, particularly in lesions with heterogeneous HBP morphology. Koh et al. also used a higher flip angle of 13–15° in the HBP compared to our 9°, which could further impact subgroup assignments.

Our analysis of prognosis centered on the visual estimation of the HBP retention area as we believed this to be more readily transferrable to clinical practice and to be better suited to characterizing heterogeneous lesions that may contain both retaining and non-retaining areas. However, this approach bears challenges as differences in HBP signal intensity between IMCCs were often subtle. There is also a lack of a clear cut-off for signal retention as IMCCs typically did not reach comparable signal intensity relative to liver parenchyma. While Koh et al. used the spleen as a reference standard for evaluating signal intensity, we gave preference to liver parenchyma because of its adjacency to the lesion, which we believed would facilitate subjective comparisons. As a result, we found only moderate agreement for estimating the Gd-EOB retention area.

Consequently, future studies that investigate the prognostic value of HBP morphology may find a quantitative approach to be more robust. Furthermore, it may be valuable to study the prognostic value of HBP morphology in conjunction with other imaging features that have been associated with the histological subtypes of IMCC. This should achieve higher specificity in inferring these subtypes, which are likely to underpin differences in prognosis between lesions. Lastly, given the tumor’s rarity, multi-center study designs will be essential to achieve adequately powered studies.

Our study has several strengths. Data on postresection histopathology and the perioperative period were available for nearly all lesions. Our cohort was also fairly large in light of this tumor’s rarity and was primarily of Western origin, unlike the Eastern-based cohorts of Koh et al. and Kang et al. The latter is an important consideration because of the higher prevalence of liver fluke infections in Asian countries, which are in turn associated with large-duct type IMCC [31]. This highlights the importance of replicating findings in regions with varying prevalences of IMCC phenotypes.

There are also limitations to our study, beginning with its retrospective nature. Second, only surgically resected IMCCs were included so that the prognostic value of imaging features in unresectable IMCCs remains unclear. Third, the follow-up period was comparatively short as a follow-up period over 3 years was only documented in one-quarter of patients. Fourth, as our significantly-retaining group was small, multivariate survival analysis could only account for a few potential confounders. Fifth, while our focus was on the prognostic value of HBP morphology, histopathological analysis to identify small-duct and large-duct IMCCs could have been of added value. Sixth, our quantitative analysis of HBP enhancement evaluated the whole tumor without taking intra-lesion heterogeneity in enhancement into account.

## 5. Conclusions

Gd-EOB-enhanced MRI identifies two imaging subtypes of IMCC in the HBP: hypointense and signal-retaining. In addition to associations with other radiologic criteria, the hypointense subtype showed significantly more frequent infiltrative growth and intrahepatic metastases. Although this finding suggests higher tumor aggressiveness, it did not translate into a significant difference in survival following primary resection.

## Figures and Tables

**Table 1:** Recorded qualitative imaging parameters, including possible values and their definitions.

Imaging Feature	Definition
Size	Largest diameter in any plane.
Enhancement: rim APHE	The lesion gain areas of hyperintensity relative to liver parenchyma in the arterial phase, primarily in the lesion’s periphery.
Enhancement: non-rim APHE	As described above, but areas of relative hyperintensity are not primarily situated in the periphery.
Enhancement: progressive	The lesion gradually enhances through the arterial, portal venous, and delayed phases, with no hyperintensity relative to liver parenchyma in the arterial phase.
Enhancement: minimal	Little or no gain in signal intensity until the HBP.
Washout: peripheral	Reduction in lesion signal intensity relative to liver parenchyma from the arterial to portal venous phase, primarily in the lesion’s periphery.
Washout: non-peripheral	As described above, but signal intensity reduction is not primarily in the lesion’s periphery.
Shape: round	Lesion shape mostly conforms to a simple sphere or oval.
Shape: lobulated	Lesion shape is primarily polycyclic.
Margin: irregular	Margins of the lesion cannot be clearly followed in post-contrast dynamic phase imaging.
Margin: sharp	Margins of the lesion are clearly demarcated in post-contrast dynamic phase imaging.
Growth pattern: solid	Lesion appears to displace and compress surrounding liver parenchyma and structures such as blood vessels.
Growth pattern: infiltrative	Lesion appears to infiltrate its surroundings, with no clear transition discernible.
Intrahepatic metastasis: local	Daughter nodules within 3 cm of the primary lesion’s border.
Intrahepatic metastasis: distant	Daughter nodules further than 3 cm from the primary lesion’s border.
Biliary dilatation	Markedly dilated biliary ducts distal to the lesion.
Macrovascular invasion	Enhancing mass within the portal vein or its major branches or hepatic veins.
Intralesional hemorrhage	Areas of hyperintensity in precontrast T1w imaging with typical morphology for hemorrhage.
Cystic components	Intralesional areas of marked, homogeneous T2w hyperintensity without enhancement.
Diffusion restriction	Intralesional hyperintensity in diffusion-weighted imaging that persists at high B-values, with low signal intensity in the corresponding ADC map.
Apparent diffusion coefficient (ADC)	Measured by placing a 2D circular ROI in a hypointense area of the lesion in the corresponding ADC map.
T1w and T2w lesion signal intensity relative to parenchyma	Subjectively rated predominant signal intensity in precontrast T1w and T2w imaging, with and without FS, relative to liver parenchyma. Lesions with slightly but not markedly lower or higher signal intensity were rated as iso- to hypointense or iso- to hyperintense.

**Table 2:** Radiological features compared between groups. Continuous variables are reported as medians, with the lower and upper quartiles in brackets. Frequencies of categorical variables are reported as percentages, with the exact proportion in brackets. APHE = arterial phase hyperenhancement. * Denotes a variable with a statistically significant difference between groups.

Radiological Features	Hypointense (*n* = 47)	Significantly-Retaining (*n* = 19)	*p*-Value
Size (mm)	67 (55–100)	63 (54–90)	0.223
Shape			0.022 *
Lobulated	100% (47/47)	89% (17/19)	
Round	0% (0/47)	11% (2/19)	
Margin			0.006 *
Irregular	83% (39/47)	47% (9/19)	
Sharp	17% (8/47)	53% (10/19)	
Growth pattern			0.005 *
Infiltrative	70% (33/47)	32% (6/19)	
Solid	30% (14/47)	68% (13/19)	
Local intrahepatic metastasis	53% (25/47)	26% (5/19)	0.039 *
Distant intrahepatic metastasis	23% (11/47)	0% (0/19)	0.022 *
Biliary dilatation	83% (39/47)	74% (14/19)	0.368
Macrovascular invasion	21% (6/28)	6% (1/18)	0.380
Intralesional hemorrhage	2% (1/47)	5% (1/19)	0.490
Cystic components	23% (11/47)	16% (3/19)	0.741
Diffusion restriction	92% (22/24)	89% (8/9)	0.443
Apparent diffusion coefficient	1094 (924–1248)	1075 (962–1181)	0.910
Enhancement pattern			0.890
Rim APHE	16% (7/45)	21% (4/19)	
Non-rim APHE	18% (8/45)	11% (2/19)	
Progressive enhancement	53% (24/45)	53% (10/19)	
Minimal enhancement	13% (6/45)	16% (3/19)	
Washout			0.151
Peripheral	2% (1/45)	16% (3/19)	
Non-peripheral	16% (7/45)	11% (2/19)	
No	82% (34/45)	73% (14/19)	
Gd-EOB retention area			N/A
0 (0–5%)	19% (9/47)	0% (0/19)	
1 (5–25%)	81% (38/47)	0% (0/19)	
2 (25–50%)	0% (0/47)	42% (8/19)	
3 (50–75%)	0% (0/47)	58% (11/19)	
4 (75–100%)	0% (0/47)	0% (0/19)	
HBP enhancement ratio	0.47 (0.39–0.63)	0.70 (0.54–0.89)	0.002 *
T1 signal intensity			0.678
Hypointense	89% (42/47)	84% (16/19)	
Iso- to hypointense	11% (5/47)	16% (3/19)	
Isointense	0% (0/47)	0% (0/19)	
Iso- to hyperintense	0% (0/47)	0% (0/19)	
Hyperintense	0% (0/47)	0% (0/19)	
T1-FS signal intensity			0.492
Hypointense	85% (40/47)	79% (15/19)	
Iso- to hypointense	15% (7/47)	21% (4/19)	
Isointense	0% (0/47)	0% (0/19)	
Iso- to hyperintense	0% (0/47)	0% (0/19)	
Hyperintense	0% (0/47)	0% (0/19)	
T2 signal intensity			0.224
Hypointense	0% (0/47)	0% (0/19)	
Iso- to hypointense	0% (0/47)	0% (0/19)	
Isointense	0% (0/47)	0% (0/19)	
Iso- to hyperintense	49% (23/47)	32% (6/19)	
Hyperintense	51% (24/47)	68% (13/19)	
T2-FS signal intensity			0.744
Hypointense	0% (0/47)	0% (0/19)	
Iso- to hypointense	0% (0/47)	0% (0/19)	
Isointense	0% (0/47)	0% (0/19)	
Iso- to hyperintense	21% (10/47)	16% (3/19)	
Hyperintense	79% (37/47)	84% (16/19)	

**Table 3:** Patient characteristics including clinical and prognostic features compared between groups. Continuous variables are reported as medians, with the lower and upper quartiles in brackets. Frequencies of categorical variables are reported as percentages, with the exact proportion in brackets. *p*-values assigned for the variables “Recurrence-free survival” and “Overall survival” derive from analysis of Kaplan–Meier curves. ° Histological grading was defined according to Edmondson–Steiner. ^ “Other complications” includes anastomotic stenosis, postoperative bleeding, portal venous thrombosis, and bile leakage without a need for intervention. # *p*-value derived from univariate Kaplan–Meier survival analysis. ICU = intensive care unit. RFS = recurrence-free survival. OS = overall survival.

Clinical Features	Hypointense (*n* = 47)	Significantly-Retaining (*n* = 19)	*p*-Value
Male gender	47% (21/47)	53% (10/19)	0.616
Age (years)	63 (55–70)	69 (58–77)	0.068
Laboratory values			
CA19-9 (U/mL) (*n* = 29)	47.8 (5.8–90.3)	24.3 (10.0–822)	0.966
CEA (µg/L) (*n* = 18)	1.6 (1.2–3.2)	2.0 (1.2–21.2)	0.500
AFP (µg/L) (*n* = 20)	4.9 (2.5–6)	5.5 (3.1–9.3)	0.628
Total bilirubin (mg/dL) (*n* = 55)	0.5 (0.3–0.8)	0.6 (0.3–1.2)	0.330
Histological grade °			0.865
1	4% (2/46)	0% (0/18)	
2	72% (33/46)	67% (12/18)	
3	24% (11/46)	33% (6/18)	
T-stage			0.305
1	37% (17/46)	37% (7/19)	
2	48% (22/46)	32% (6/19)	
3	11% (5/46)	16% (3/19)	
4	4% (2/46)	16% (3/19)	
N1 or higher	51% (23/45)	41% (7/17)	0.565
V1-status	15% (7/46)	26% (5/19)	0.299
R1-status	24% (11/46)	41% (7/17)	0.322
Postoperative complications			
Any complication	72% (31/43)	88% (15/17)	0.311
Liver failure	10% (3/31)	15% (2/13)	0.617
Kidney failure	7% (3/42)	12% (2/17)	1.000
Pneumonia	16% (7/43)	24% (4/17)	0.481
Intra-abdominal abscess	14% (6/43)	29% (5/17)	0.261
Anastomotic insufficiency	9% (4/43)	29% (5/17)	0.100
Bile leakage requiring intervention	19% (8/43)	29% (5/17)	0.486
Other complications ^	26% (11/43)	35% (6/17)	0.547
Postoperative hospital stay (days)	13 (8–23)	18 (13–49)	1.000
Postoperative ICU stay (days)	2 (1–3)	1 (1–5)	0.617
Recurrence-free survival			0.410 #
Time to recurrence (days)	204 (94–415)	217 (62–498)	0.830
3-month RFS	82% (32/39)	60% (12/20)	0.662
6-month RFS	71% (24/34)	50% (11/22)	1.000
12-month RFS	59% (17/29)	43% (9/21)	0.719
18-month RFS	45% (10/22)	33% (6/18)	0.240
Overall survival			0.491 #
Time to death (days)	518 (113–809)	464 (255–805)	0.612
3-month OS	97% (38/39)	100% (14/14)	0.662
6-month OS	87% (34/39)	100% (14/14)	1.000
12-month OS	69% (27/39)	93% (13/14)	0.719
18-month OS	56% (22/39)	71% (10/14)	0.240

**Table 4:** Multivariate Cox regression for the analysis of RFS and OS dependent on visually assessed significantly-retaining appearance or quantitatively assessed HBP enhancement ratio in combination with potential further radiological and postresection histopathological predictors of survival. Lesions with hypervascular appearance show rim or non-rim APHE. Adjusted hazard ratios are visualized in a forest plot on a logarithmic scale in the right-adjacent column; 95% confidence intervals for estimates of hazard ratios are given in brackets. * Denotes a variable with a statistically significant difference between groups. HR = hazard ratio.

Factors	Adjusted HR, RFS		*p*-Value	Adjusted HR, OS		*p*-Value
Model 1		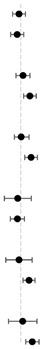			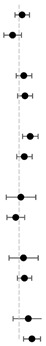	
HBP signal-retention	0.84 (0.44–1.61)		0.602	1.38 (0.66–2.89)		0.397
Hypervascular appearance	0.70 (0.36–1.36)		0.297	0.52 (0.21–1.27)		0.151
Model 2						
HBP signal-retention	1.26 (0.62–2.53)		0.526	1.64 (0.74–3.67)		0.226
Intrahepatic metastasis	2.53 (1.34–4.75)		0.004 *	1.85 (0.86–3.96)		0.116
Model 3						
HBP signal-retention	1.05 (0.52–2.32)		0.891	3.16 (1.43–6.97)		0.168
N1 or higher	2.85 (1.50–5.40)		0.001 *	1.73 (0.79–3.79)		0.004 *
Model 4						
HBP enhancement ratio	0.74 (0.19–2.87)		0.667	1.21 (0.27–5.51)		0.802
Hypervascular appearance	0.72 (0.35–1.46)		0.356	0.72 (0.29–1.77)		0.468
Model 5						
HBP enhancement ratio	0.84 (0.22–3.15)		0.790	1.59 (0.36–7.04)		0.545
Intrahepatic metastasis	2.31 (1.26–4.23)		0.007 *	1.76 (0.85–3.62)		0.127
Model 6						
HBP enhancement ratio	1.22 (0.29–5.11)		0.784	2.61 (0.54–12.5)		0.231
N1 or higher	3.22 (1.64–6.32)		0.001 *	3.77 (1.58–8.97)		0.003 *
		0.1 1 10			0.1 1 10	

## Data Availability

The datasets used and/or analyzed during the current study are available from the corresponding author upon reasonable request.

## Supplementary Materials

The following supporting information can be downloaded at: https://www.mdpi.com/article/10.3390/cancers16071314/s1, Table S1: Representative MRI acquisition parameters for Gd-EOB-enhanced MRI at 1.5 T.
